# Comparison of Novel Coronary Artery Disease Risk Factors between Obese and Normal Adolescent

**Published:** 2015-07

**Authors:** Samaneh Kouzehgaran, Rahim Vakili, Mohsen Nematy, Mohamad Safarian, Majid Ghayour-Mobarhan, Mohamad Khajedaluee

**Affiliations:** Research Center and Department of Nutrition, Faculty of Medicine, Mashhad University of Medical Sciences, Mashhad, Iran

**Keywords:** Obesity, Overweight, Coronary artery disease, Adolescents

## Abstract

**Background:**

Coronary artery disease is considered as the most common cause of death in all societies including Iran. This study seeks to compare the new risk factors of coronary-artery diseases in obese adolescents and control group.

**Methods:**

In this cross-sectional study, amongst the obese adolescents registered in the nutrition clinic of Ghaem Hospital, 80 individuals were selected. As the control group, additional 80 adolescent students having the same gender and age as the obese group, but with normal weight were selected. These two groups were selected randomly and their serum level of vitamin D, anti-heat shock protein27 (HSP27), balance of oxidants and antioxidants, and homocysteine were determined and compared.

**Results:**

In this study, 42 (53.2%) and 37 (46.8%) of the obese and normal weight groups were male, respectively. The mean value of triglyceride, cholesterol, and LDL in the obese group was higher than the normal group, but the mean value for HDL, vitamin D, homocysteine, PAB (Preoxidant and Antioxidants Balance), and anti-HSP27 was not significantly different between the groups. In the base of homocysteine >15 µmol/l, 26.6% of the obese group had hyperhomocysteinemia, therefore homocysteine may be a new risk factor for coronary artery disease in obese adolescents (χ^2^=4.072; P value=0.091).

**Conclusion:**

The findings of this study showed that despite the presence of obesity in adolescence and adolescents, new risk factors are not present among them more than the control group. This was in contrast to what was seen in adults.

## Introduction

Coronary artery disease is the most common cause of death in all societies including Iran. Despite advances made in the identification of risk factors and the mechanisms that cause this disease, it is not thoroughly preventable.^1^ In the US alone, coronary heart diseases have been responsible for more than 450,000 deaths in 2004, and more than 13 million people suffer from this disease.^2^

Various risk factors, including smoking, hypertension, high cholesterol, diabetes, and obesity have been identified as the causes of coronary artery disease.^3^

Traditional risk factors in different studies are assessed and the effect of traditional risk factors in adolescents is clear. However, new factors are recently identified as CAD risk factor.

Today, there are a growing number of people getting this disease without having major risk factors. In a study undertaken by Hajimoradi et al., the mean level of homocysteine in 37 patients suffering from coronary artery stenosis and 35 patients without coronary artery stenosis was reported as 18.88±9.5 (µmol/Lit) and 13.68±4.88 (µmol/Lit), respectively.^4^

Nowadays, new risk factors such as lipoprotein A, fibrinogen, homocysteine, and inflammatory markers such as CRP (C-reactive protein) have posed themselves as the potential risk factors of coronary artery disease. Moreover, extensive studies conducted on the role of vitamin D, HSPs, and oxidant and antioxidant balance in heart disease have produced varied results. For instance, the results of some studies revealed that homocysteine not only was an important independent risk factor for coronary artery disease, but it was also a strong predictor of the mortality in reported patients.^5^

In a study conducted in Asia in 1997, previous risk factors were reported responsible for only 50% of coronary artery disease.^5^ Similarly, in a case-control study carried out by Samani et al. on patients suffering from coronary artery disease, the mean value for total homocysteine was significantly higher than the control group (19.25±8.2 and 14.8±4.17, respectively).^6^

A study was performed in India to recognize the importance of homocysteine as a risk factor for coronary artery disease (CAD) in young patients. They demonstrated that there was an independent association of this new risk factor with traditional ones in young CAD patients.^7^

The correlation between coronary artery disease with pro oxidant/antioxidant balance and oxidative DNA damage was investigated in a study by Kaya et al. They concluded that, pro-oxidant/antioxidant balance and oxidative stress play an important role in the pathogenesis of CAD.^8^

Another factor that has been the subject of recent studies as a risk factor for coronary artery disease is vitamin D deficiency. Some studies have shown that vitamin D prevents the development of myocardial hypertrophy and cardiac dysfunction and treatment with vitamin D results in reduced cardiac hypertrophy and QT in hemodialysis patients, which is an important risk factor in sudden cardiac death.^9^

The vascular oxidative stress and the imbalance between oxidant and antioxidant have been identified as another risk factor in heart disease. Studies revealed that chronic production of oxygen free radicals in pathophysiologic conditions had a pivotal role in the development of heart disease.^10^

Oxygen free radicals bring about various events such as oxidation of LDL, migration, and proliferation of endothelial and smooth muscle cells. Oxidative stress acts as the reinforcing mechanism for many risk factors in cardiovascular disease.

As other possible risk factors for cardiovascular disease and atherosclerosis, HSPs are also explored in this plan. Physiological function of HSPs is to protect cells against apoptosis, but if this protein is stimulated pathologically by these risk factors, it leads to a series of inflammatory and proliferative responses in smooth muscle cells and macrophages, which eventually bring about the production of antigens against cells and the development of atherosclerosis process.^11^

Changes in lifestyle, such as indoor jobs, wearing occlusive clothing and extensive use of sunscreen creams, have increased the number of individuals with low vitamin D levels. There is an inverse association between serum 25(OH)D and metabolic syndrome and cardiovascular disease.^12^

Suffering from coronary artery disease without any major risk factor, especially at an early age, necessitates the investigation of other risk factors, especially in people who do not exhibit any other major risk factors. In Iran, we do not have enough information about the new coronary artery disease risk factors in obese adolescents. Therefore, we conducted this study to compare the concentrations of homocysteine vitamin D, HSP’s, and oxidant and antioxidant balance in obese with normal adolescents and assessed new risk factors in the Iranian adolescent population. 

## Materials and Methods

This study was performed in the clinic of nutrition in a tertiary academic hospital in Mashhad during one year (2010-2011). In this cross-sectional study, adolescents who were willing to participate in the study and had a BMI above 95^th^ percentile were selected; their age was between 12-17 years. They also required meeting certain criteria such as the absence of any precedent of medicine consumption (except for acetaminophen and cold pills) or disease (except for the common cold or transient viral disease) and being a known case of AIDS and hepatitis B/C in order to be included in this study. Informed consent was obtained from the studied population. Eighty individuals were selected with the same gender and age (12-17 years) with normal BMI from Mashhad high schools and assigned to the control group. Weight was measured without shoes and wearing only light clothing using an electronic weighing scale (Rassa, Tehran, Iran) and recorded to the nearest 100 grams. Height was measured once at baseline without shoes with the individuals stretching to the maximum height and the head positioned in the Frankfort plane using a portable stadiometer (OTM, Tehran, Iran) recorded to the nearest 0.1 cm. Body mass index (BMI) was also calculated (kgm^–2^).

To determine the level of vitamin D, homocysteine, oxidant and antioxidant balance, and anti-HSP 27 in the population, 5cc venous blood was taken from all individuals and sent to the laboratory under standard conditions. 

The Prg kits (Sigma & Merck Co.) were used to determine the level of vitamin D. The standard solutions were prepared by mixing varying proportions (0–100%) of 250 μM hydrogen peroxide with 3 mM uric acid (in 10 mM NaOH) and TMB powder (Merck Co., Germany). Peroxides from horseradish practical Grade and Chloramin T (AppliChem, Germany) were used to measure PAB (oxidant and antioxidant balance). 

Serum Hsp27 antibody titres were measured using an in-house ELISA assay. Microtitre plates (NUNC MaxiSorp, Nottingham, UK) were coated with 100 ng per well recombinant human Hsp27 dissolved in 50 micro liter carbonate buffer, pH 9.6 incubated for 18 h at 4ºC under humidified conditions. The wells were washed three times in wash buffer (PBS containing 0.05% Tween-20).

A full fasted lipid profile, comprising of the total cholesterol, triglycerides, high-density lipoprotein cholesterol (HDL-C), and low-density lipoprotein cholesterol (LDL-C), was determined for each patient. Serum lipid concentrations were measured by enzymatic methods. The data were analyzed using SPSS 15 software. Center, dispersion, and frequency distribution parameters were utilized in descriptive statistics. In order to compare the qualitative and quantitative variables, Chi-Square test for comparing and Kolmogorov-Smirnov test for assessing the normality were used. The sample size was calculated with the formula: (β=95 and α=0.01)

$$N=-\frac{\left({2.65+1.64)}^{2}(s_{1}^{2\;}+s_{2}^{2\;\;}\right)}{{\left(x_{1\;}-x_{2\;}\right)}^{2}}$$

$$\frac{{\left(2.65+1.64\right)}^{2\;\;}(10^{2\;}+33^{2})}{{\left(14-30.5\right)}^{2}}=82$$

If the normal distribution assumption was achieved t-test, otherwise Mann-Whitney test was used. In all cases, P≤0.05 was considered as the level of significance. 

## Results

Hundred obese adolescents were enrolled in this study and 20 were excluded due to the use of specific drugs. In this study, 42 (53.2%) and 37 (46.8%) of the obese and normal weight groups were male, respectively. There were no differences in variable sex between the groups (χ^2^=0.054; P=0.816). Kolmogorov-Smirnov test showed that all quantitative variables in this study were not normally distributed.

Average body mass index (BMI) in the obese and normal groups was 31.57±2.75 and 20±2.27, respectively. The mean values of triglyceride, cholesterol, and LDL were 99.53±37.66, 158.61±30.24, and 99.36±23.47, respectively. The mean value of triglyceride, cholesterol, and LDL in the obese group was higher than the normal group, but the mean values for HDL, vitamin D, homocysteine, PAB, and anti-Hsp27 were not significantly different between both groups (tables 1 and 2).

**Table1:** Comparison of outcome measures between control and case groups

**Variable**	**Group**	**n**	**Mean±SD**	**Mann-Whitney test**
BMI (kg/m^ 2 ^)	Obese group	79	31.57±2.75	P<0.001*
	Normal group	80	20±2.27	
Triglyceride (mg/dl)	Obese group	79	105.64±40.19	P=0.041*
	Normal group	80	93.49±34.16	
Cholesterol (mg/dl)	Obese group	79	166.03±30.78	P=0.001*
	Normal group	80	151.28±28	
HDL (mg/dl)	Obese group	79	36.85±6.05	P=0.119
	Normal group	80	36.45±5.45	
LDL (mg/dl)	Obese group	79	107.47±27.19	P<0.001*
	Normal group	80	91.34±15.52	

*Significant at 0.05

**Table 2:** Comparison of outcome measures between control and case groups

**Variable**	**Group**	**n**	**Mean±SD**	**Mann-Whitney test**
Vitamin D (mg/ml)	Obese group	79	28.68±10.75	P=0.863
	Normal group	80	31.27±17.84	
Homocysteine (µmol/Lit)	Obese group	79	13.12±5.79	P=0.091
	Normal group	80	11.64±6.2	
PAB (HK unit)	Obese group	79	125.56±23.8	P=0.427
	Normal group	80	129.32±19.48	
Anti Hsp27 (µg/ml)	Obese group	79	0.3±0.22	P=0.609
	Normal group	80	0.28±0.21	

*Significant at 0.05

The percentage of patients with homocysteine higher than 15 μmol/Lit was 26.6% in the obese group and 13.8% in the normal group, respectively. Chi-Square test showed a significant association between homocysteine and groups under the investigation and it may be a new risk factor for coronary artery disease in obese adolescents (χ^2^=4.072; P=0.091). The blood triglyceride levels for 9 persons (11.4 %) in the obese group and 4 persons (5%) in the control group were higher than normal; the relationship between cholesterol, HDL, LDL, and vitamin D and groups were not significant in this study (table 3).

**Table 3:** Comparison of outcome measures between control and case groups

** Group** **Variable**	**Obese group N (%)**	**Normal group N (%)**	** χ^ 2 ^ (P value) **
Abnormal triglyceride (mg/dl)	9 (11.4)	4 (5.0)	2.163 (0.141)
Abnormal cholesterol (mg/dl)	19 (24.1)	6 (7.5)	8.217 (0.004)*
Abnormal HDL (mg/dl)	58 (73.4)	57 (71.3)	0.093 (0.760)
Abnormal LDL (mg/dl)	17 (21.5)	0 (0.0)	19.276 (<0.001)*
Abnormal homocysteine (µmol/Lit)	21 (26.6)	11 (13.8)	4.072 (0.049)*
Abnormal vitamin D (mg/ml)	0 (0.0)	0 (0.0)	-

*Significant at 0.05; **The value of abnormal (TG >150, LDL >130, cholesterol >190, HDL <40, homocysteine >15, and vitamin D <10)

## Discussion

Our study demonstrated that in obese adolescents, new risk factors for coronary artery disease are not present which was in contrast to what we observed in adults. 

The results of this study revealed that, the mean value of vitamin D level in the normal group (31.27) was higher than the obese group (28.68), though, it was not statistically significant (P=0.86). Furthermore, the mean value of homocysteine level in the obese group (13.12) was higher than the control group (11.64), but it was not significant (P=0.091). In a case-control study that compared 51 patients under 45 years of age with 15 healthy individuals; Puri et al. reported homocysteine as an independent risk factor for young patients suffering from coronary artery disease and recommended the examination of the homocysteine for all young patients in the absence of any typical risk factors.^13^

In a study by Sierakowska-Fijalek on 48 adolescent with atherosclerosis risk factors, homocysteine level was significantly higher than healthy adolescents in the control group.^14^ We had similar finding and the level of homocysteine was higher in obese adolescents than those in the normal group, but it was not significant.

Salari et al. suggested in their study that, extracellular HSP27 acts as a signaling molecule to activate NK-kB (a key immune signaling modulator in atherogenesis) in macrophages.^15^

Egerton et al. measured the serum homocysteine level in patients hospitalized for MI in two time points, once on the MI day and the other after 180 days. The results indicated that at the first time point, the average homocysteine level was 12.9±0.09 while in the second time point it was 15.3±1. This difference has been substantiated by other case-control studies as well. In the current study, the triglyceride and cholesterol levels in the obese group were significantly higher than the control group, though the mean value for HDL and LDL did not reveal any significant difference between the groups.^16^ In our study, the triglyceride (P=0.041) and cholesterol (P=0.001) levels in the obese group was significantly higher than the control group too.

In a study carried out by Glowinska et al., the cholesterol concentration in all adolescents under the investigation was higher (166.3±33 mg/dl) than the control group (153.4±23 mg/dl), which is consistent with the findings of the current study. In the above-mentioned study, the LDL concentration of the case group (97.3±29 mg/dl) was higher than the control group (89.7±23 mg/dl). This value was lower than that of our study. Moreover, in the above-mentioned study, the concentration of HDL in the study groups was 47.4±14, which was not significantly different from that of the control group (i.e. 47.9±12). In that study, HDL level was higher than ours was.^17^

In a study conducted by Hamidi et al., the balance of oxidant and antioxidant in the control and cardiovascular disease group was 49±41.4 and 115.9±39.7 respectively, showing a statistically significant difference between the two groups.^18^ In the present study, the average balance of oxidants and antioxidants in the obese and control groups did not show any difference (P=0.42).

The findings of the study by Hamidi et al. indicated that the effect of oxidative stress forms an important risk factor of atherosclerosis and monitoring the balance of oxidant and antioxidant along with other risk factors can serve as a useful laboratory-clinical test in the diagnosis of cardiovascular events.^18^

In our study, the mean level of anti-HSP27 in the obese and control groups was 0.3 and 0.27 respectively, which revealed no statistically significant difference between the two groups. The findings of this study showed that, despite the presence of obesity in adolescence and adolescents, new risk factors are not present among them more than the control group. It is probably due to the fact that, these factors need time to affect or perhaps different defense mechanisms in adolescents could be the reason. 

One limitation of our study is the number of study groups; another limitation is that we did not have the dietary pattern in these groups. 

## Conclusion

The findings of this study showed that, despite the presence of obesity in adolescence and adolescents, new risk factors are not present among them more than the control group. This was in contrast to what was seen in adults. 
